# Nasal administration of diacetylmorphine improved the adherence in a patient receiving heroin-assisted treatment

**DOI:** 10.1186/s12954-022-00644-2

**Published:** 2022-06-07

**Authors:** Maximilian Meyer, Jean N. Westenberg, Johannes Strasser, Kenneth M. Dürsteler, Undine E. Lang, Michael Krausz, Marc Vogel

**Affiliations:** 1 University Psychiatric Clinics Basel, University of Basel,, Basel, Switzerland; 2 Department of Psychiatry, University of British Columbia, Vancouver, Canada; 3 Department of Psychiatry, Psychotherapy and Psychosomatics, Psychiatric Hospital, University of Zurich, Zurich, Switzerland

**Keywords:** Heroin-assisted treatment, Diacetylmorphine, Nasal administration, Opioid agonist treatment, Opioid use disorder

## Abstract

**Background:**

Traditional heroin-assisted treatment in Switzerland consists of oral and injectable diacetylmorphine (pharmaceutical heroin) administration. To date, no suitable treatment option is available for patients who crave rapid onset (“rush”) but are either unable to inject or primarily sniff or inhale illicit heroin. We present a patient who successfully switched to intranasal heroin-assisted treatment following several unsuccessful treatment attempts.

**Case presentation:**

A 29-year-old male with severe opioid use disorder, injection substance use, and concomitant cocaine use, previously prescribed slow-release oral morphine, was started on intravenous diacetylmorphine. Due to complications and harms associated with intravenous injections, nasal diacetylmorphine was prescribed. With this novel route of administration, the patient who had previously been unable to adhere to other OAT options remained in treatment. Health outcomes improved by reduction of injection-related harms, increased adherence to the heroin-assisted treatment regimen, and increased collaboration with the therapeutic staff.

**Conclusions:**

Nasal heroin-assisted treatment can be a feasible therapeutic option for individuals with severe opioid use disorder who crave the fast onset of effect of diacetylmorphine but are unable to inject intravenously.

## Background

Some individuals with opioid use disorder (OUD) respond poorly to traditional opioid agonist treatment (OAT) such as methadone, slow-release oral morphine (SROM), or buprenorphine for a variety of reasons (e.g., persisting cravings, lack of "rush" or "high", unmet opioid requirements). For these patients, repeated oral treatment attempts without significant benefit can result in decreased treatment adherence, increased use of illicit opioids, and increased risk of poor health and social outcomes. In order to reach these individuals with therapeutic interventions that better suit their needs, heroin-assisted treatment (HAT) was introduced in Switzerland in 1994 [1], which consists of oral and/or injectable diacetylmorphine (DAM; pharmaceutical heroin). The prescribed supervised use of DAM is currently regulated in several European countries and Canada, and has been proven clinically effective for individuals who would otherwise remain outside of the healthcare system [2–4]. In Switzerland, HAT is provided in specialised outpatient treatment centres as well as one prison and is subject to strict legal regulations [5, 6]. To meet the eligibility criteria for HAT, patients must be 18 years or older, have had OUD for at least two years, need to have previously failed two treatment attempts, and present with psychological, physical, or social harms caused by their opioid dependence [7]. Prescription DAM is provided within supervised clinical settings, which allow monitoring for safety and ensure compliance. Take-home medication can only be prescribed to select stable patients or under exceptional conditions (e.g., COVID-19 isolation [8]). HAT therefore demands of patients a strict regular schedule that allows them to attend the HAT clinic once or twice daily for DAM administration. This can be a barrier for some patients who are prevented from consistently frequenting the HAT clinic or taking the medication reliably due to social and structural factors (e.g., homelessness, transportation difficulties, long travel distances, unstructured lifestyles, mental or physical comorbidities).

In Switzerland, DAM has only been approved for intravenous (IV) and oral administration [1], which poses a substantial barrier for individuals who mainly inhale or snort street heroin. Inhalable DAM was initially evaluated in the 90s by prescribing heroin-cigarettes (reefers, “sugarettes”) to patients. However, reefers only yielded a small amount of DAM and showed little effect in treatment settings, resulting in these prescriptions being stopped [9]. According to recent data from the European Monitoring Centre for Drugs and Drug Addiction (EMCDDA), snorting is the main route of administration for 25% of heroin users entering treatment, whereas inhaling accounted for an additional 41% [10]. These patients can often not be recruited for or retained in HAT as the available DAM formulations do not fit these patients’ needs. For patients who primarily use the nasal route of administration, the prescription of injectable HAT raises serious ethical concerns due to the higher risks associated with this route of administration. The use of oral DAM as an alternative does not provide the same psychotropic effects when compared to other more rapid onset routes of administrations (e.g., intravenous, nasal) due to the comparatively prolonged time to peak plasma concentration [11–13].

In addition, the average age of individuals in OUD treatment is increasing in Switzerland and in parts of Europe [14, 15]. The most recent EMCDDA data suggests that high-risk drug users above 40 years old may soon become the largest drug treatment population in Europe [16]. This population is at high risk of developing injecting-related injuries and diseases like scarred veins, infections of the blood vessels and heart valves, abscesses, and other soft-tissue infections [17].

Swiss HAT centres have been evaluating nasal HAT (n-HAT) as a suitable alternative to injectable or oral DAM among patients who do not have a history of injection substance use and primarily use the nasal route of administration, as well as for patients who are no longer able to inject intravenously or for whom continued injections constitute a severe health risk [18]. We present a patient who was started on IV DAM because traditional oral OAT programmes were not addressing his need for the fast onset of effect. However, he was unable to attend his IV DAM administration appointments regularly and lacked safe injection practices (heavy bleeding, repeated unsuccessful injections, disregard of hygienic precautions). He was transferred to IN DAM which aligned with his substance use treatment goals, improved retention in care, improved his health and social outcomes, and supported his stabilisation. Written informed consent from the patient was obtained for participation and for publication of the case report.

## Case presentation

In September 2021 a 29-year-old male was referred for HAT at our specialised treatment centre. He presented with heroin use disorder, cocaine use disorder, and trichotillomania. Previous medical documentation stated a psychiatric history of attention deficit disorder and combined personality disorder. He had first used heroin at the age of 17, started snorting the substance regularly at the age of 25, and transitioned to daily injection use at the age of 27. The patient has a history of injecting opioids in an unsanitary manner (lots of blood stains following injection) and has received multiple bans from several different harm reduction facilities (e.g., safe injection rooms) due to this. He did not suffer from injection related chronic infections such as HIV or Hepatitis C. His previous OUD treatment history included slow-release oral morphine (SROM) at adequate doses, which did not enable him to cease high-risk use behaviour. Despite receiving OAT, he still regularly injected illicit substances due to the rapid onset of effects provided through IV administration. At his HAT intake appointment, which provides an opportunity for the medical team to meet the patient, outline the therapeutic framework, assess patient's past psychiatric and substance use history, and conduct a medical examination, he emphasised a strong desire to start IV DAM and was deemed eligible for HAT. He was to continue on his SROM regimen until the start of HAT, after which he would receive injectable DAM twice daily and prescribed SROM as take-home medication if necessary.

His first IV DAM administration was scheduled two weeks after his intake appointment. This is the norm, as approval for the treatment must be obtained from the Swiss Federal Office of Public Health before DAM can be prescribed. As by our centre rules, first-ever DAM administrations require scheduled appointments so that patients can be supervised by the physician in charge.

**Fig. 1:**
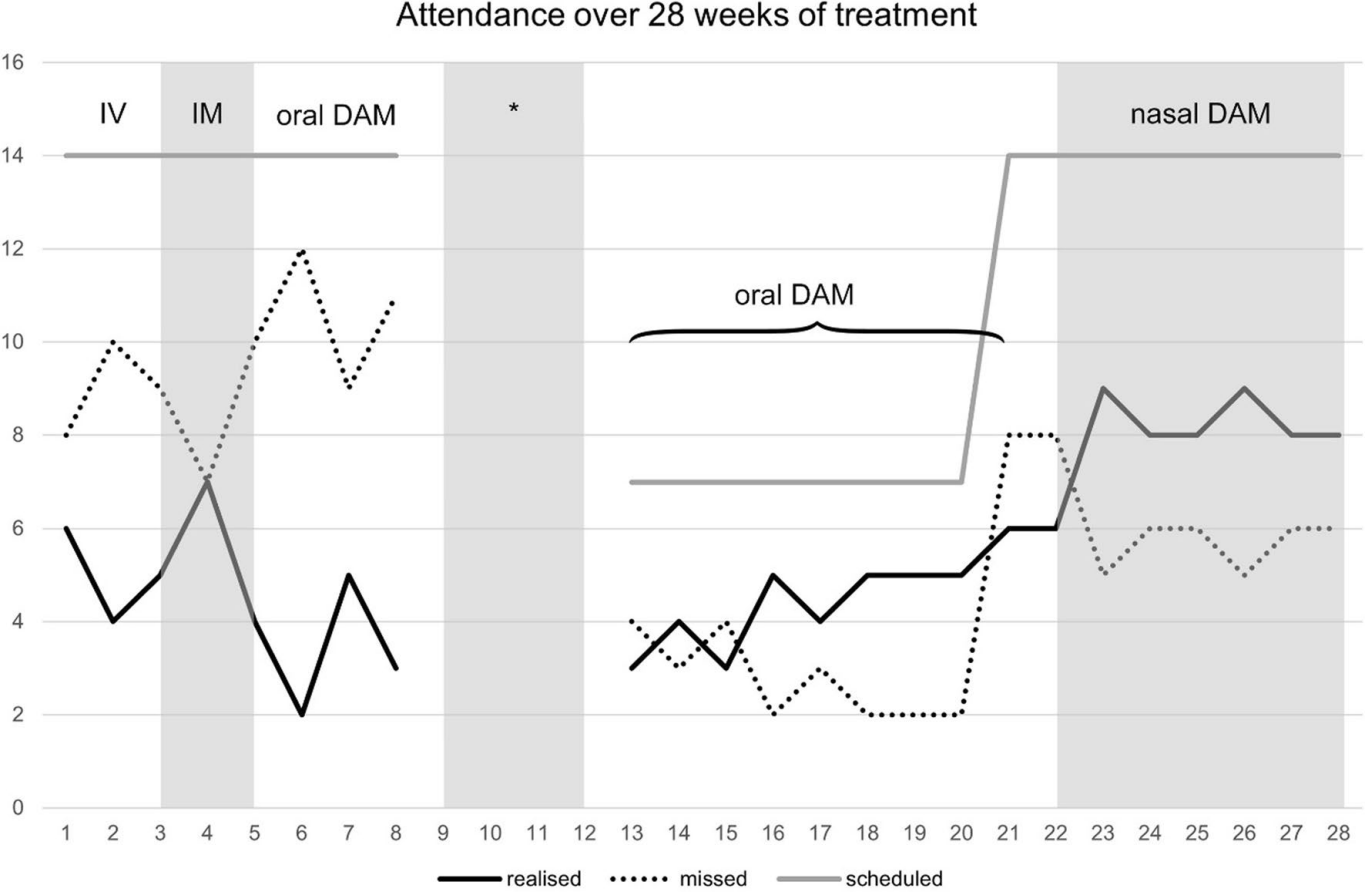
Scheduled, realised, and missed DAM administrations over the course of 28 weeks. IV: intravenous; IM: intramuscular; *Referred to another treatment centre due to violation of HAT centre safety policies

In the first week of treatment, it was clear the patient was having trouble following the strict therapeutic framework of HAT, as he missed eight of 14 scheduled DAM administrations for the week (Fig. 1). Moreover, when administering DAM, the patient also demonstrated unsafe injection practices. He had trouble finding suitable access veins, did not disinfect sites prior injection despite repeated requests of nurses, accidentally contaminated the application room with blood after injection, and continued to impulsively pierce his skin without palpating suitable veins. As a result, his IV DAM administrations often took approximately 45 min, either until he found a suitable vein, or until he proceeded to injecting intramuscularly (IM), which corresponds to a common practice in HAT despite off-label use. Small abscesses and erythema on his arms were also evident, which he stated were due to his IV use of street heroin and cocaine. We therefore asked the patient whether he preferred switching to n-HAT, but declined, insisting on continuing to inject. Hence, we prescribed the IM administration of DAM, but the patient was dissatisfied with the effects that IM DAM provided and continued to miss multiple consecutive DAM administrations. Due to repeated missed scheduled DAM administrations, our treatment protocols required DAM to be restarted in low doses as opioid tolerance could no longer be safely assumed.

In the sixth week of treatment, oral DAM was prescribed because the patient was continuing to try injecting intravenously and did not follow the safe injection regimen that is enforced at the HAT centre. He went on to miss 12 of 14 scheduled oral DAM administrations (Fig. 1) and expressed the urge to continue trying to inject intravenously. Indeed, on the 8th week of treatment, the patient dissolved and injected an SROM capsule in the lavatory of the HAT centre. He was therefore asked to discuss the incident with the physician-in-charge but became verbally aggressive and left the premises. As is the norm in the case of severe non-adherence to the centre's safety guidelines, the patient was referred to a different outpatient treatment centre that only offered traditional OAT options for a 2-week period (“time out”). Upon his return on the first day following this 2-week period, the patient entered the HAT centre and reached into the used syringe container in an effort to find syringes with remaining DAM from other patients. Due to this impulsive and aggressive behaviour, which could have caused harm to himself or others, the patient was once again referred to an outpatient OAT centre for another 2-week period.

Following this second 2-week period, the patient was restarted on oral DAM, but continued to miss most of his scheduled administrations (Fig. 1). He continued to express a wish to begin IV DAM, stating that the reason for not attending regularly was that the tablets were not addressing his needs for a rapid onset of effects (“rush”). He also continued to present with skin infections (erythema and abscesses) in the crook of both arms due to intravenous use of illicit substance.

In order to align with the patient's wishes, on the 21st week of treatment, we prescribed injectable DAM additionally to the existing oral prescription, under the condition that he would only inject under the supervision of the physician-in-charge. Despite being provided with instructions during his IV administrations, he continued to have difficulties performing his administrations safely, often taking over an hour, and disinfecting his injection sites only if explicitly reminded to do so. He missed more than half of his scheduled IV DAM administrations during this week (Fig. 1).

During the 22nd treatment week, he was offered two alternative treatment options: returning to oral DAM or starting IN DAM. He opted for IN DAM, despite having previously ruled this out as a treatment option, likely because he already knew that oral DAM would not fit his opioid needs. Followingly, he was prescribed 340 mg IN DAM twice daily. The patient received sterile DAM solution and administration was made possible by providing syringes with a screw-on atomizer instead of a needle. The IV dose was converted to IN DAM with the assumption that the bioavailability of morphine following nasal administration lies in-between the bioavailability of oral and IV administrations. After the first IN DAM administration, he reported being pleasantly surprised by the subjective effects. He expressed interest in continuing the IN DAM prescription. The patient started attending more frequently (Fig. 1) for the first time since initiation of treatment and consistently realised more scheduled DAM administrations than he missed. For the first time in his treatment trajectory, he had not needed to restart his DAM dosing due to missed appointments and possible loss of opioid tolerance. This made it possible to further increase his IN DAM dose in 30 mg increments until the patient stated the dose (two times 430 mg) to fit his needs. After 4 weeks of IN DAM (week 26), the patient’s skin showed a significant improvement (no erythema or abscesses). From a subjective perspective, he expressed that even though the effects of IN DAM were not as strong as IV, they were sufficient to reduce his craving for fast-acting illicit opioids. He stated being happy to see the progression of his skin healing and felt proud of being able to heavily reduce injections of illicit substances. He also stated relief and being under less pressure during the nasal administration process. This was because he had to spend less time in the administration room as the repeated and exhaustingly long search for suitable access veins was no longer necessary.

## Discussion and conclusions

This case report presents the successful clinical use of n-HAT for a patient with severe OUD for whom traditional oral treatment options (including oral DAM) were insufficient due to the much slower onset of effect and for whom IV treatment options resulted in deteriorated vein status, chronic ulcerations, and intramuscular or risky body part-injections. To our knowledge, there is only one other published case-series on n-HAT, in which we similarly illustrated the feasibility of supervised n-HAT in a clinical therapeutic setting [18]. Our case report adds to the emerging literature by demonstrating the ability of n-HAT to retain a patient in care who had previously been unable to adhere to an OAT treatment regimen, improve health outcomes by reducing harms related to injection substance use, increase adherence to the strict HAT therapeutic framework, and increase collaboration with the HAT centre staff.

The patient presented in this case report started on oral slow-acting OAT (SROM), then transitioned back-and-forth between injectable and oral DAM, before successfully being retained on intranasal DAM. This trajectory demonstrates the benefits of focusing on the patients’ unique goals, needs and preferences, which align with the principles of patient-centred care and harm reduction [19]. More individualised treatment options are needed in order to overcome the “one-size-fits-all” approach prevalent in many OUD treatment settings [20]. IN DAM was an essential treatment option for retaining the patient in care, and clinical trials are undeniably required to appropriately and firmly evaluate n-HAT in OUD treatment.

The strict therapeutic framework of HAT which is time-intensive and requires a structured daily routine for a regular, often twice daily, attendance presents a barrier to many patients with OUD who qualify for HAT. This often results in missed DAM administrations, which can sometimes necessitate restarting the dosing regimen, as was the case for this patient. Incrementally increasing the DAM dosing after a prolonged period of missed scheduled DAM administrations, done out of precaution and patient safety, was frustrating for the patient and aggravated his dissatisfaction with the HAT regimen. This also led to a cycle, as the starting DAM doses were too low, which increased the patient's likelihood to miss a scheduled administration and consequently barred any dose increase if administrations were in fact missed. In the case of this patient, n-HAT was able to break this cycle, allowing the patient to achieve a dose that met his opioid requirements.

Intranasal HAT also addresses the adverse harms associated with illicit IV substance use or—to a minor extent—prescribed IV DAM. Currently, patients that are no longer able to inject DAM into peripheral veins due to deterioration of the veinous system may revert to injecting in risky body parts (e.g., veins in the groin [21]), intramuscular or subcutaneous injection [22], or change to oral DAM tablets, a pattern reflected in our case report [14]. All of these alternatives have been associated with complications such as infections and abscesses, indurations or skin lesions and are often described as painful, particularly subcutaneous and intramuscular injections, which ultimately reduce treatment outcomes [23, 24].

This case report demonstrates the therapeutic potential of n-HAT. Multiple advantages of IN administration are illustrated by this case, when compared to other routes of administration. Firstly, it improved the patient’s health outcomes by reducing the likelihood of physical health complications (e.g., infection, lesions) associated with injection behaviours. Secondly, it provided stronger psychotropic effects when compared to oral DAM and intramuscular DAM, which underlines that some patients require a rapid onset of opioid effects which is associated with the subjective experience of a “rush” or “high”. Thirdly, it is very low threshold without requiring stringent hygiene protocols and is not dependent on the patient’s success in finding a vein, which benefits both the patient, the healthcare team, and the limited resources of the HAT centre.

This case report, along with a previously published case-series, highlights the feasibility of n-HAT as a novel route of administration for HAT and a possibly viable alternative to injectable or oral HAT. More long-term research efforts are needed to systematically assess the efficacy and acceptability of n-HAT among individuals with severe OUD.

## Abbreviations

OUD: Opioid use disorder
OAT: Opioid agonist treatment
SROM: Slow-release oral morphine
DAM: Diacetylmorphine
EMCDDA: European Monitoring Centre for Drugs and Drug Addiction
HAT: Heroin-assisted treatment
IV: Intravenous
IN: Intranasal
n-HAT: Nasal heroin assisted treatment
IM: Intramuscular
